# Bartholin’s gland cyst caused by *Sneathia amnii*: a case report

**DOI:** 10.1186/s12879-023-08302-z

**Published:** 2023-05-17

**Authors:** Ranran Zhang, Zhezhong Zhang, Minjing Xu, Wenjie Li, Yanwen Sun, Yuliang Dai, Xuejing Yang, Shaohua Lin

**Affiliations:** Department of Clinical Laboratory, The First Affiliated Hospital of Zhejiang Chinese Medical University, 54 Youdian Road, Hangzhou, 310006 Zhejiang P.R. China

**Keywords:** *Sneathia amnii*, Bartholin’s gland cyst, Fastidious bacteria, Bacterial vaginosis, Case report

## Abstract

**Background:**

*Sneathia amnii* is a conditional pathogen of the female genital tract that is involved in bacterial vaginosis and poor reproductive and perinatal outcomes. Few studies have reported subcutaneous cysts following invasive infection caused by *S amnii*.

**Case presentation:**

Here we report the case of a 27-year-old woman who presented with Bartholin’s gland cyst due to *S amnii* infection, and was successfully treated with surgical neostomy and antibiotic agents. The isolate was gram-negative, bacillary, anaerobic, and was identified by polymerase chain reaction (PCR) amplification of the 16 S rRNA.

**Conclusions:**

*S amni* is an important but underappreciated pathogen that needs further investigation. This report describes the microbial and pathogenic characteristics of *S amnii* and is expected to provide a valuable reference in obstetric and gynecologic clinical practice.

## Background

*Sneathia amnii*, initially named *Leptotrichia amnionii*, was first isolated from the amniotic fluid of women after intrauterine fetal demise [1]. *S amnii* is a gram-negative, bacillary, anaerobic, fastidious pathogenic bacterium that requires nutrient-rich media [2]. Due to the constraints of complex nutrient requirements and slow growth mode, molecular methods are mainly used to identify and diagnose *S amnii* infection. The presence of *S amnii* was associated with preterm labor, chorioamnionitis, stillbirth, peripartum bacteremia, bacterial vaginosis, sexually transmitted diseases, and other invasive infections [3]. Studies showed that *S amnii* could traverse the tissue and cell barriers, and spread within infected tissues by its virulent determinants and pathogenicity [4]. We report a case of *S amnii* as the causative agent of Bartholin’s gland cyst and have reviewed the literature on this pathogen.

## Case report

A 27-year-old, previously healthy female was hospitalized because of swelling and pain in her vulva. The mass, which was located on the left labia majora, was red, warm, and tender. The patient had not received any treatment before visiting the doctor. On admission, the patient’s blood pressure was 118/68 mmHg; heart rate, 95 beats/min; and body temperature, 38℃. Laboratory findings suggested inflammation (C-reactive protein, 38.90 mg/L) and hyperleukocytosis (14.1 × 10^9^/L). The pH of leucorrhea was elevated (pH, 4.8) and the amine test was positive. Cervical secretions tested negative for human papillomavirus (HPV). Serologic tests for syphilis and human immunodeficiency virus were both negative. Ultrasonography showed a cystic mass (measuring approximately 5.6 × 3.6 cm) in the subcutaneous area of the vulva with poor ultrasonic echo. The condition was diagnosed as Bartholin’s gland cyst for which we decided to perform surgical neostomy and cystectomy. The patient was placed in the lithotomy position after anesthesia, and the vaginal mucosa of the inner labia majora was dissected. The purulent fluid in the cyst was extracted for microbiological examination and then washed repeatedly with povidone-iodine. The stoma was formed by discontinuous suturing between the cyst wall and vaginal mucosa. The patient was administered clindamycin phosphate injection and supportive treatments postoperatively and she recovered after 6 days.

## Microbiological analysis

**Fig. 1:**
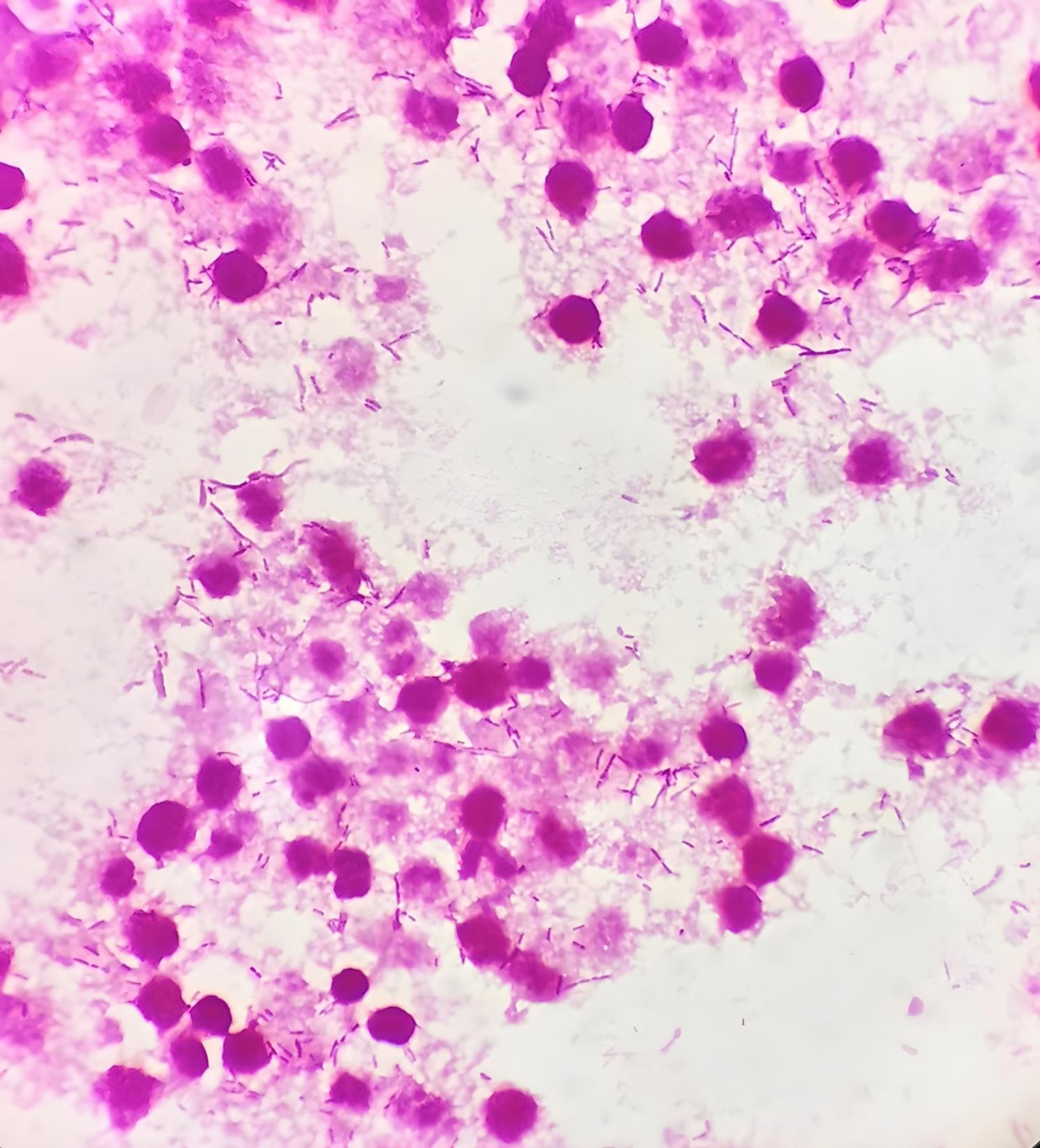
Gram stain of *Sneathia amnii* colonies from positive blood culture bottle showing long and short gram-negative rods (image at ×1000 magnification)

The purulent fluid was cultured on Columbia blood agar, MacConkey agar, and chocolate agar under aerobic conditions (35 °C, 5% CO_2_). Meanwhile, 2 ml purulent fluid was inoculated into BacT/Alert PF (pediatric FAN blood culture bottle) and incubated in the BACT/ALERT 3D system (BioMérieux, Marcy l’Etoile, France). No bacterial growth was observed in agar after the 48-hour culture. However, bacterial growth was observed in the blood culture bottle after 5 h of incubation. Gram staining of the positive bottle showed long and short gram-negative rods (Fig. 1). The cultures from the positive blood culture bottle were inoculated onto Columbia blood agar and incubated under aerobic and anaerobic conditions. Gray and translucent colonies sized < 1 mm were seen after 72 h of incubation under anaerobic conditions. Conversely, there was no bacterial growth on the aerobic blood agar. However, the isolate was not identified by MALDI-TOF spectrometry mass (Bruker Daltonics, Massachusetts, USA). The molecular approach was considered to identify the pathogenic bacteria. The isolate was identified by polymerase chain reaction (PCR) amplification of the 16 S rRNA, and species identification was based on the consistency of 16 S rDNA sequences in the National Center for Biotechnology (NCBI) database. The isolate displayed 99.8% sequence similarity to the NCBI database sequence for *S amnii*.

## Discussion

*S amnii* is an important but underappreciated pathogen affecting women’s health because of its complex nutritional requirements, slow growth patterns, and requirement of anaerobic conditions. *S amnii* causes bacterial vaginosis and poor reproductive and perinatal outcomes. *Sneathia* species (spp.) have been identified as members of Community State Type IV (CST-IV) of the vaginal microbiome, which is characterized by the decrease of *lactobacillus* spp. in the vagina and elevated pH of vaginal secretions [5]. *Sneathia* spp. were also found in the male urogenital tract, and may get transmitted to the sexual partners [6]. Studies revealed that the overgrowth of *S amnii* was observed in HPV-positive women, and apparently, as a consequence of an HPV disease/cervical cancer [7]. Furthermore, *S amnii* in septic complications of pregnancy was involved in the pathogenesis of septic abortion, intrauterine fetal demise, and stillbirth [1, 8, 9]. Several cases of *S amnii* infections outside the genital tract have been described. Eugen et al. described an *S amnii* (*L amnionii*) infection in the genitourinary tract of renal transplant recipients [10]; Carina M et al. described a renal abscess in a female 5 weeks postpartum with *S amnii (L amnionii)* bacteremia [11]; *S amnii (L amnionii)* also caused bacterial arthritis in a male patient undergoing hemodialysis [12], and spondylitis in a female patient with endometrium adenocarcinoma [13]. Here, we have described a Bartholin’s gland cyst caused by *S amnii* in a female with bacterial vaginosis. These results demonstrated that *S amnii* may transmit invasive infection from the lower reproductive tract to the other tissues.

To our knowledge, this is the first description of an *S amnii*-infected Bartholin’s gland cyst in a patient with bacterial vaginosis. The pH of vaginal discharge in this patient was elevated (pH, 4.8) and the test for amines was positive. A previous study revealed that *Snathia spp.* were more frequently present in vaginal secretions from women showing symptoms of bacterial vaginosis compared with those in asymptomatic women; moreover, the clue cells (vaginal epithelial cells with bacteria on the surface) were observed in the vaginal microbiome having an overgrowth of *Sneathia spp.* [14]. The Bartholin’s gland cyst caused by *S amnii* infection may be correlated with the cytotoxicity and pathogenicity of this pathogen. Gabriella L et al. have reported that *S amnii* has strong hemolytic properties and was capable of damaging cells within the fetal membranes [4]. Moreover, the virulence of *S amnii* was associated with a pore-forming cytotoxin CptA that destabilizes host cell membranes [4]. Additionally, Michael et al. have reported that *S amnii* releases hydrolytic enzymes to cross the barriers of tissue and membranes [15]. These results demonstrated that *S amnii* with cytotoxicity and pathogenicity could pass cell barriers and spread within infected tissues. However, there are limited studies discussing the mechanisms of virulence and pathogenicity of *S amnii*.

Here, *S amnii* was identified from positive blood culture bottles and isolated on Columbia blood agar after anaerobic culture for more than 48 h, and identified by 16 S rRNA gene amplification and sequencing. Bartholin’s gland cyst and inflammatory symptoms caused by *S amnii* infection have been successfully treated with surgical neostomy and clindamycin phosphate.

For the treatment of Bartholin’s gland cyst, empirical antibiotics can be administered before the pathogen was identified when combined with systemic infection symptoms, such as fever. Usually, quinolones combined with metronidazole were recommended for anti-infection. *S amnii* may cause upward transmission of the invasive infection from the lower reproductive tract to other tissues. Therefore, clinicians should pay more attention to the significant role of *S amnii* in women’s health and disease; further studies on *S amnii* infection are warranted.

## Data Availability

All data generated or analysed during this study are included in this published report.

## Abbreviations

*S amnii*: *Sneathia amnii*
*L amnionii*: *Leptotrichia amnioni*
HPV: human papillomavirus
PCR: polymerase chain reaction
16 S rRNA: 16 S ribosomal ribonucleic acid
NCBI: National Center for Biotechnology
